# Zinc as a Modulator of miRNA Signaling in Obesity

**DOI:** 10.3390/nu17213375

**Published:** 2025-10-28

**Authors:** Nurpudji Astuti Taslim, Anne Maria Graciela, Dante Saksono Harbuwono, Andi Yasmin Syauki, Andrew Nehemia Anthony, Nur Ashari, Andi Makbul Aman, Raymond Rubianto Tjandrawinata, Hardinsyah Hardinsyah, Agussalim Bukhari, Fahrul Nurkolis

**Affiliations:** 1 Division of Clinical Nutrition, Department of Nutrition, Faculty of Medicine, Hasanuddin University, Makassar 90245, Indonesia; 2 Faculty of Medicine, Hang Tuah University, Surabaya 60239, Indonesia; 3 Division of Endocrinology, Metabolism, and Diabetes, Department of Internal Medicine, Faculty of Medicine, Universitas Indonesia, Dr. Cipto Mangunkusumo National Referral Hospital, Jakarta 10430, Indonesia; 4 Department of Internal Medicine, Faculty of Medicine, Hasanuddin University, Makassar 90245, Indonesia; 5 Center for Pharmaceutical and Nutraceutical Research and Policy, Atma Jaya Catholic University of Indonesia, Jakarta 12930, Indonesia; 6 Division of Applied Nutrition, Department of Community Nutrition, Faculty of Human Ecology, IPB University, Bogor 16680, Indonesia; 7 Master of Basic Medical Science, Faculty of Medicine, Universitas Airlangga, Surabaya 60131, Indonesia; fahrul.nurkolis.mail@gmail.com; 8 Institute for Research and Community Service, State Islamic University of Sunan Kalijaga (UIN Sunan Kalijaga), Yogyakarta 55281, Indonesia; 9 Medical Research Center of Indonesia, Surabaya 60286, Indonesia

**Keywords:** zinc, microRNA, obesity, insulin resistance, adipogenesis, inflammation, epigenetics, nutritional genomics, precision nutrition, biomarker

## Abstract

**Background**: Obesity is a multifactorial metabolic disorder influenced not only by excessive caloric intake but also by micronutrient imbalances such as zinc deficiency. Emerging evidence suggests that zinc regulates microRNA (miRNA) biogenesis and expression, linking nutritional status to metabolic regulation. **Objective**: This review delineates the molecular interplay between zinc and miRNAs in obesity, emphasizing the mechanistic, clinical, and translational relevance of zinc-sensitive miRNAs in adipogenesis, insulin resistance, inflammation, and oxidative stress. **Results**: Zinc deficiency alters miRNA expression profiles associated with metabolic dysregulation. Key miRNAs—miR-21, miR-34a, miR-122, and miR-144-3p—are consistently modulated by zinc status, influencing inflammation, lipid metabolism, and insulin signaling. Zinc repletion restores several miRNAs (e.g., miR-10b, miR-155, miR-145), suggesting reversibility, while excessive zinc may upregulate miR-144-3p and exacerbate oxidative stress. Circulating and exosomal miRNAs show promise as dynamic biomarkers for zinc intervention efficacy. **Methods**: A literature search was performed in 4 databases up to August 2025 using keywords related to zinc, miRNAs, and obesity. Eligible studies included both preclinical and human research evaluating zinc status or supplementation and miRNA expression in metabolic contexts. **Conclusion**: Maintaining optimal zinc levels may normalize miRNA expression and improve insulin sensitivity. The “zinc–miRNA axis” represents a novel frontier for precision nutrition in obesity management.

## 1. Introduction

The obesity epidemic has reached an alarming state, contributing to the increase in metabolic disorders such as type 2 diabetes, cardiovascular disease, and nonalcoholic fatty liver disease. Although obesity is traditionally viewed as a consequence of overeating and decreased activity, evidence has emerged indicating the important role of micronutrient deficiencies, particularly zinc, in the pathophysiology of obesity and its related health conditions [1,2]. On a molecular level, a link between zinc, microRNAs (miRNAs), and metabolic signaling networks is emerging [1,3].

Zinc is involved in numerous biological activities related to enzymatic reactions, immunity, and antioxidation [1]. Recent evidence suggests that zinc also participates in miRNA biogenesis and function and, as such, may be an important intermediary between nutrition and gene expression [4]. The recognition of miRNAs as significant modulators of numerous pathways, including adipogenesis, insulin sensitivity, and inflammatory response, suggests that understanding how zinc impacts miRNA function may bring a new perspective and lead to the development of novel therapeutic targets for obesity and its related diseases [3].

The current literature provides evidence that zinc status influences the expression of obesity-related miRNAs in tissues such as adipocytes and liver [2,5]. Some key miRNAs in obesity, such as miR-21, miR-34a, and miR-122, have been shown to be responsive to zinc [2,3]. Additionally, Alder et al. [5] and Beckett et al. [2] found that zinc nutritional status is associated with altered miRNA profiles in circulation as well as in tissues, indicating zinc’s regulatory role in miRNA-mediated systemic changes associated with obesity and metabolic disease. Although considerable advances have been made in this area, more information is still needed regarding the roles of individual zinc transporters, such as ZIPs and ZnTs, and their regulation by specific miRNAs, particularly in regard to tissues and cells targeted by zinc signaling [6]. More information is needed to better translate the emerging miRNA–zinc connection in obesity to nutritional and clinical recommendations [2].

Despite extensive evidence linking zinc deficiency to metabolic dysfunction, few studies have comprehensively explored the molecular interplay between zinc homeostasis and miRNA-mediated regulatory networks in obesity. This review uniquely integrates mechanistic, clinical, and translational evidence to elucidate how zinc acts as a molecular switch influencing miRNA biogenesis, expression, and function in adipose and metabolic tissues. Unlike previous reviews that examined zinc or miRNAs separately, our synthesis highlights zinc-responsive miRNAs—such as miR-21, miR-34a, miR-122, and miR-144-3p—as critical mediators connecting micronutrient signaling to adipogenesis, insulin resistance, and inflammation. The primary objective of this paper is to delineate the bidirectional regulation between zinc and miRNAs in obesity, summarize current preclinical and clinical findings, and propose a mechanistic framework for their translational application in nutritional metabolic research, also highlight opportunities for precision nutrition strategies that target the zinc–miRNA axis to prevent or mitigate obesity-related metabolic disorders.

### Search Strategy and Study Selection Criteria

A comprehensive literature search was conducted to identify studies that examined the relationship between zinc, microRNA (miRNA) regulation, and obesity. The databases PubMed/MEDLINE, Scopus, Web of Science, and ScienceDirect were searched for relevant articles published up to August 2025. Publications from 1 January 2014 to 31 August 2025 were included, covering an eleven-year span that captures both foundational and up-to-date evidence on zinc–miRNA interactions in obesity and metabolic regulation. The search strategy combined both Medical Subject Headings (MeSH) and free-text terms, including “zinc,” “Zn,” “zinc supplementation,” “zinc deficiency,” “microRNA,” “miRNA,” “miR,” “obesity,” “metabolic syndrome,” “insulin resistance,” “adipogenesis,” and “metabolic disorders.” To ensure a comprehensive capture of the available evidence, the reference lists of retrieved articles and relevant reviews were also manually screened for additional studies. Only articles published in English and available in full-text form were considered.

The inclusion of studies was based on their relevance to the mechanistic, experimental, or clinical interplay between zinc and miRNA expression within the context of obesity and metabolic regulation. Eligible studies comprised both preclinical models (in vitro and in vivo) and human research that evaluated the effects of zinc status or supplementation on miRNA expression linked to adipogenesis, insulin signaling, oxidative stress, or inflammation. Studies that did not directly assess the zinc–miRNA relationship, lacked molecular or metabolic outcome data, or focused primarily on other trace elements or nutrients without miRNA involvement were excluded from the review.

All identified records were screened for relevance by two independent reviewers through title, abstract, and full-text examination, with any discrepancies resolved by discussion and consensus. From the included publications, data regarding study design, biological model or population, zinc intervention or exposure, target miRNAs, analytical methods such as qRT-PCR or sequencing, and reported metabolic outcomes were extracted. The final selection of studies provided a comprehensive overview of the molecular and clinical evidence supporting the role of zinc as a modulator of miRNA signaling pathways in obesity and related metabolic diseases, forming the foundation for the thematic synthesis presented in this review.

## 2. Zinc in Metabolism and Disease

The maintenance of metabolic equilibrium is significantly influenced by zinc, owing to its effects on cellular transport and enzymatic operations. Systemic health, in addition to the onset of metabolic conditions like diabetes and obesity, is subject to its regulation [1,7]. A comprehension of these complex mechanisms yields critical perspectives into the ways in which micronutrient dynamics are connected to molecular pathways, which is consistent with the wider initiatives to investigate individualized strategies for the management of metabolic diseases [1,8], as delineated in this review.

### 2.1. Zinc Transport Mechanisms

**Figure 1:**
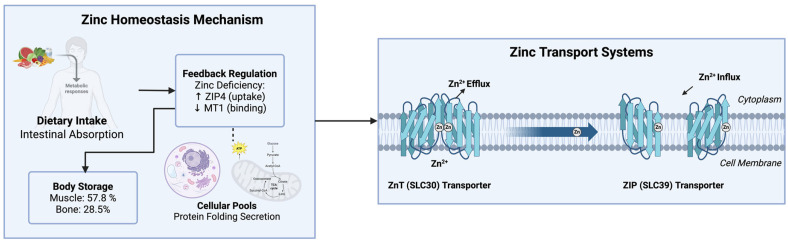
Zinc homeostasis and transport systems in the human body. Dietary zinc is absorbed in the intestine, stored in muscle and bone, and regulated via ZIP (SLC39) and ZnT (SLC30) transporters to maintain cellular balance. ↑ (increase), ↓ (decrease).

Zinc transport in the body is carried out by two primary families of transporters: ZnT (SLC30) and ZIP (SLC39) [6]. ZnT transporters pump zinc out of the cytosol into an organelle or to the extracellular milieu (Figure 1), whereas ZIP transporters promote zinc influx into the cytosol [1,6]. Given the diverse roles of zinc in multiple biological pathways and its cofactor functions, the regulation of its transport, along with the metabolism and regulation of its transporters, are crucial (Figure 1). The metabolic consequences caused by the disruption of zinc transporter function by mutation, dysfunction, or misregulation provide insight into the importance of their proper regulation, which is highlighted by the association of altered zinc transporter regulation with metabolic disorders [6,9]. For instance, it has been found that zinc transport can be modulated during the metabolic syndrome and that its alterations impinge on zinc-dependent cellular processes [1,6].

The proteins contained in the early secretory pathway (endoplasmic reticulum (ER), Golgi apparatus and ER-Golgi intermediates) depend on zinc transporters for the acquisition of zinc for their catalytic activities or structural integrity. The disruption of zinc transport in the early secretory pathway leads to the induction of ER stress, resulting in improper protein folding, impaired protein maturation, and secretion. ER stress interferes with the normal metabolic regulatory network of cells, inducing the progression of metabolic diseases. Therefore, the effects of the individual zinc transporters on the ER must be evaluated to understand their contribution to normal or pathological ER homeostasis [6,10].

Zinc is stored mostly in the muscles (57.8%), bone (28.5%), skin (4.9%), and liver (4.6%). Approximately only 4% of the total zinc body content is distributed among all of the other tissues. The cellular pools of zinc are located in the nucleus, cytosol, organelles, membranes, and vesicles. All of these transport demands must be met by the zinc transporters, whose activity is likely responsive to variations in the compartmentalized distribution of zinc at the intracellular and systemic levels [1,7].

Intestinal zinc absorption is highly sensitive to feedback regulation in situations where there is dietary zinc deficiency. In such situations, the intestinal ZIP4 transporter is upregulated for zinc uptake, whereas metallothionein-1 (MT1), a zinc-binding protein that regulates intracellular zinc levels, is downregulated, ensuring a sufficient amount of zinc uptake [11,12]. Variations in zinc transporters are involved in multiple diseases, as indicated by the findings that a mutation in *SLC30A8*, which encodes *ZnT8*, affects type 2 diabetes risk, and that the knockout of *ZIP13* influences beige adipocyte development. Such studies provide evidence for the impact of the regulation of zinc transporters on obesity and metabolic syndrome [9,13].

Obesity is typically associated with decreased serum zinc and abnormal zinc transporter expression, both leading to impaired immunity and an increased inflammatory response. Experimental results indicate that zinc transporter activity, by controlling the cellular zinc concentration, is associated with a polarized macrophage response and inflammatory pathways during inflammation. This demonstrates that obesity impairs immunity and promotes inflammation due to altered zinc transporter expression [1,14].

In summary, the zinc transport systems are important homeostatic regulators of the metabolism and the delivery of zinc across cells and in the body. These mechanisms are likely associated with cellular growth and homeostasis through numerous zinc-dependent cellular events such as genetic regulation, protein maturation, and immune responses [1]. Zinc transporters, through regulation of the proper delivery and localization of zinc, appear to have an important role in the physiology of many metabolic pathways, and alterations in their function result in pathological effects, which can lead to chronic metabolic diseases like obesity and type 2 diabetes [9].

### 2.2. Metabolic Functions

Zinc is a necessary cofactor of over 300 enzymes and several transcription factors. Zinc plays a crucial role in different processes such as DNA replication, gene expression and cell signaling. The significance of zinc for many metabolic processes indicates a need for the optimization of its homeostasis to avoid any damage. When the function of the zinc-dependent enzymes is disrupted due to limited amounts of zinc, this can be reflected as metabolic dysfunction [1,15,16]. As such, zinc can be a marker in metabolic vulnerability [1,17]. The question that is left to be answered is whether the function of individual zinc-dependent enzymes differs between experimental and dietary conditions [6].

The maintenance of metabolic tissue function is regulated by the function of the zinc transporters such as the ZIP (SLC39) and ZnT (SLC30) families, which regulate the flux of zinc into cellular organelles such as the ER and Golgi [6,18]. The significance of ER and Golgi lies in their importance for the protein folding and secretion of enzymes with a need for zinc. Due to the importance of zinc transporters in organelle homeostasis, its malfunction can lead to the ER stress situation where a lot of proteins are folded inappropriately. These are often seen in obese people and the individuals with metabolic syndrome. Given the importance of protein transport, these organelle zinc transporters can be thought to be an important regulatory step for protein folding that regulates cellular adaptive responses to metabolism [1,6].

Zinc has several modulatory effects on insulin signaling and glucose metabolism via certain enzymes and pathways. It can regulate the activation of insulin receptor kinase, stimulate insulin binding to its receptor and affect enzymes involved in downstream signaling [1,19]. Studies indicate a consistent relationship between low dietary zinc intake and metabolic syndrome, and supplementation trials also suggest that adequate zinc intake may be beneficial for glycemia and reducing plasma glucose levels [17,19]. Thus, intervention studies and the prevalence of metabolic syndrome can suggest the preventive and therapeutic roles of zinc [1,19].

The interaction between zinc and the plasma proteome in diseases, such as obesity, can alter inflammation and coagulation. The effect of zinc depletion on the expression of pro-inflammatory proteins such as fibrin β, which contributes to coagulation, can induce certain changes in experimental results. Fibrin β protein levels are significantly increased when zinc is depleted and can reverse with zinc repletion [17,20]. These outcomes indicate that zinc can be an early regulator of disease progression associated with inflammation and obesity.

The regulatory role of zinc as an anti-inflammatory agent extends into a broader nutritional setting as it can also alter metabolism-related pathway signaling in response to different dietary factors and even regulate the expression of miRNAs, reflecting context-dependent actions of zinc [2,21]. One example of context-dependent regulation involves zinc’s ability to increase the potency of polyphenols on the modulation of antioxidant defense. Polyphenols are generally known to increase zinc’s bioavailability in the body. Dietary factors such as fatty acids can also have a profound impact on the context-dependent role of zinc in metabolic homeostasis. All these interactions with dietary factors suggest the role of zinc is intertwined with the nutritional milieu in regulating metabolism, and they can alter a lot of metabolic processes that are regulated by zinc as an anti-inflammatory element [1,21]. In conclusion, we have to be conscious of the presence of other dietary bioactives to identify the role and impact of zinc on metabolic health.

The epigenetic regulation and expression of miRNAs can modulate gene function with no direct interaction of gene sequence [3,4]. Some of the mechanisms which these regulatory actions involve are DNA methylation and histone acetylation [4,22]. Zinc also regulates the process by which small, non-coding RNAs called miRNAs are made by the enzyme, called Dicer. Thus, zinc can alter epigenetic changes to alter miRNA processing. Given the essential role of miRNA regulation in obesity and metabolic syndrome, it can be suggested that the effect of zinc supplementation is directly linked to miRNA regulation [2,3]. miRNAs play an important role in obesity and metabolic syndrome by regulating adipogenesis, inflammation and insulin resistance, so can they be used as potential targets for the treatment and prevention of obesity and metabolic syndrome.

In summary, the metabolic function of zinc depends on its impact on multiple interactions: enzymatic functions, nutrient interactions, cellular signals and epigenetics. The regulation by zinc on metabolic pathways indicates a critical need to ensure the proper systemic zinc homeostasis to prevent disruption of metabolic mechanisms. Although the effects of zinc in metabolic diseases are being understood more and more, its intricate role and the benefits of zinc as a nutritional intervention are still not fully understood. More research should be conducted to determine the appropriate intake and mechanisms to improve metabolism and metabolic processes within an individual.

## 3. miRNA Regulation in Obesity

MicroRNAs (miRNAs) are critical mediators of gene expression, regulating various aspects of adipogenesis, insulin signaling, and inflammation during obesity [3,4]. Through a network of targets and tissue-specific functions, they appear central to metabolic health and disease. Investigation into the key miRNAs, their targets, and their roles in disease pathogenesis is essential for further exploration into diagnostic and therapeutic applications and for connecting the molecular mechanisms of zinc–miRNA interactions to the broad aspects of zinc status and obesity, reviewed here.

### 3.1. Key miRNAs and Their Targets

MicroRNAs are critical post-transcriptional regulators of gene expression influencing multiple cellular pathways, including adipogenesis, insulin signaling, lipid metabolism and chronic inflammation in the context of obesity and metabolic dysfunction [3,23]. For example, miR-21 regulates inflammation and adipogenesis by directly suppressing tumor suppressor gene *PDCD4*, while miR-34a inhibits *SIRT1*, a key player in mitochondrial biogenesis. Similarly, miR-122 modulates lipid synthesis by affecting cholesterol and fatty acid metabolic pathways [3,24]. Understanding the regulatory role of these miRNAs, their mechanism of action in obesity and metabolic syndrome, as well as upstream factors governing their expression, should facilitate the development of effective diagnostic and therapeutic strategies.

Obesity-associated miRNAs interact with broader networks, influencing multiple gene targets in the adipocyte as well as other tissues that regulate central metabolism. The gene target of miR-21 is *PDCD4*, a tumor suppressor protein associated with chronic inflammation and fibrosis [3,5]. On the other hand, miR-34a inhibits *SIRT1*, the critical regulator of mitochondrial biogenesis and metabolic health. miR-122 modulates systemic cholesterol and fatty acid metabolism. It has been demonstrated that miR-122 reduces circulating LDL-C, elevates plasma TG and increases hepatic fat, suggesting its impact on whole-body energy homeostasis [25]. While the individual roles of miRNAs in cellular metabolism have been described, the overall redundancy and specificity of their targets, their interaction with global metabolic networks in obesity and their response to a variety of stimuli, such as nutrients, inflammation, hormones and so forth, remain to be further investigated [3].

miRNAs act as metabolic messengers and regulators responding to changes in the internal as well as external environment [2,3]. This bi-directional activity implies that miRNAs play a key role in metabolic responsiveness in obesity. As such, a critical component of understanding the function of obesity-related miRNAs is to determine how their expression is regulated by physiological parameters and pathophysiological conditions. It is known that high-fat diet or overnutrition alters miRNA levels, causing subsequent perturbation in their target pathways [3,26,27]. More detailed studies are necessary to address this bi-directional relationship to discern how miRNAs, altered by environmental factors, influence metabolism. It needs to be determined if altered miRNA expression is merely a consequence of chronic overnutrition, or if in fact it contributes to metabolic disequilibrium.

While miRNAs can be tissue specific, certain miRNAs are present in multiple cell types, regulating expression pathways across tissues [3,28]. For example, hepatic-expressed miR-122 can alter systemic cholesterol levels. However, miRNAs such as miR-21 and miR-34a are present in many tissues, including the liver, adipocytes and inflammatory cells. These miRNAs play crucial roles in tissue-to-tissue signaling that are likely important in the development of the systemic complications of obesity and metabolic syndrome [3,24]. Therefore, the investigation of the biological role of these obesity-related miRNAs needs to consider the complexity of cell-type specificity in relation to the overall impact on whole-body metabolic homeostasis.

Gene expression is finely controlled by regulatory sequences that are bound and/or altered by external and internal stimuli, such as nutrients, hormones, and pharmacological agents. This allows a rapid and adaptable response to changing circumstances and to maintain metabolic homeostasis. While miRNA-mediated modulation of networks represents a major step forward in regulatory understanding, it introduces significant complexity in terms of systemic consequences, such as the potential for unpredicted interactions that may have deleterious outcomes. For example, decreased levels of several miRNAs have been reported in individuals suffering from metabolic syndrome [3,4]. The role of miRNAs in the fine tuning of gene networks suggests that miRNA manipulation could improve insulin sensitivity. For example, it has been found that the expression of the miRNAs miR-130a and miR-130b is markedly reduced in plasma and adipocyte samples from obese patients [29,30].

Experiments using specific miRNA transgenic or knockout animals confirm that these molecules play an essential role in metabolic control, providing an additional avenue for the therapeutic regulation of the complications of obesity [23]. In mice, deficiency for the let-7 family of miRNAs blocks insulin resistance induced by a high-fat diet, which suggests that these are key regulators of metabolic phenotype. These results support the potential use of miRNA inhibition as a therapeutic strategy to prevent the development of diabetes in individuals who are overweight. However, more specific and precise control of miRNA regulation, through the use of antisense oligonucleotides or other tools, would reduce off-target effects. Such therapies should be based on a complete understanding of miRNA expression levels in a variety of tissues and metabolic states [3,31]. For example, levels of hepatic miR-26a are decreased in overweight and obese humans, a change that correlates to hepatic accumulation of TG and impaired insulin signaling, suggesting that miRNA alterations could contribute to obesity-related liver disease [3]. Further investigation of tissue-specific expression of miRNAs and their effects on metabolism in these tissues is needed.

It is known that chronic overnutrition, inflammation and oxidative stress, three main characteristics of obesity, influence the expression of metabolic genes. Altered miRNA profiles in obesity directly feed back on metabolic targets, altering a number of nutrient sensing, adipogenic and insulin-signaling genes. These altered patterns affect multiple steps in nutrient sensing and glucose metabolism and adipocyte regulation and promote the cycle of metabolic dysfunction in obesity. To this end, a comprehensive evaluation of the expression levels and effects of miRNAs in the context of obesity will clarify the physiological and clinical roles of miRNA and may provide novel diagnostic and therapeutic options [3,32]. It has been shown that zinc deficiency induced an elevation in the expression of oncogenic, pro-inflammatory miRNAs such as miR-21, miR-31, and miR-146a in tissues like the esophagus and tongue, which suppresses the expression of tumor suppressor genes, including *PDCD4* and PPP2R2A, thereby showing that zinc is a key nutrient for miRNA networks regulating metabolic inflammation and tissue homeostasis. Significantly, zinc repletion reverses this effect in esophageal epithelial cells, with lowered levels of oncogenic miRNAs and restoration of tumor suppressor proteins, illustrating a connection between nutritional status and miRNA-driven oncogenesis [5,33]. In particular, similar miRNA dysregulation is reported in many human cancers. Overexpression of miR-21 and miR-31 is seen in zinc-deficient tissue culture cells, which is analogous to the overexpression observed in human cancers. Furthermore, in vivo and in vitro analysis shows that miR-31 is a cell-type-specific miRNA that predominantly localizes to tumor cells. In contrast, miR-21 is expressed in stromal cells [5]. A better understanding of this cell-type specificity in relation to oncogenesis is required to develop zinc-targeting therapies to mitigate against cancer risk in human health.

For instance, zinc supplementation resulted in increased expression of miR-144-3p, which targets the gene for the antioxidant transcription factor Nrf2, consequently exacerbating oxidative stress and insulin resistance. Experimentally targeting miR-144-3p shows that it mediates cellular responses to zinc treatment, suggesting a novel target to improve antioxidant capacity during zinc treatment. These reports show that the effects of zinc on human health are more intricate than initially assumed. Therefore, detailed studies are warranted to further decipher zinc’s effects on these miRNAs [34]. A high-throughput screening approach has been used to identify several zinc-responsive miRNAs in plant systems, implicating them in several metabolic and stress-related pathways, indicating that zinc is an essential element in maintaining miRNA homeostasis. Translating results from plant research to animal systems could enhance the understanding of miRNA control of metabolic processes by zinc. These findings provide evidence that zinc, and other divalent micronutrients, control a broader network of miRNAs and that these miRNAs, in turn, control many target genes that are essential for the cellular maintenance of mineral-dependent processes [35].

Another dimension of the relationship between zinc and miRNAs is the regulation of miRNA biogenesis. Zinc regulates the expression and activity of *DGCR8* and *Drosha*. Stress-induced conditions, such as those observed in obesity, suppress both the Drosha and *DGCR8* protein levels, implying that a complex network of signaling pathways controls miRNA processing [4,36]. Overall, zinc has multiple effects on miRNA regulation that can impact on metabolic health at multiple stages.

### 3.2. Expression in Metabolic Disease

**Figure 2:**
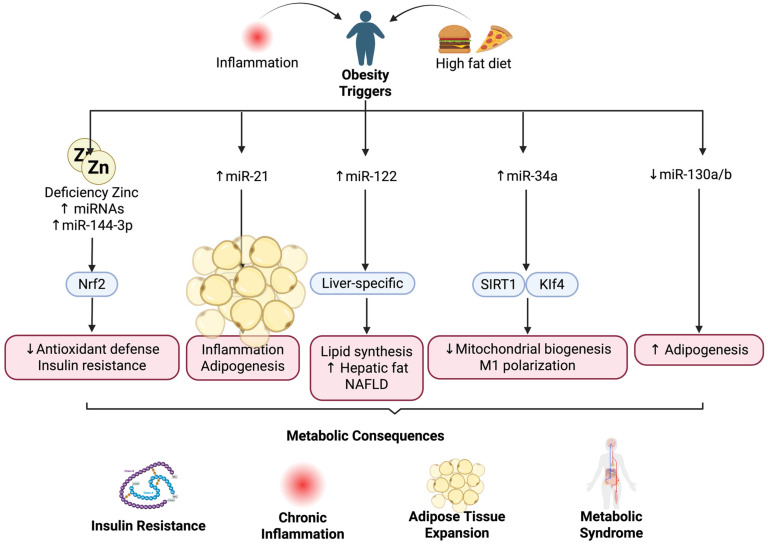
Zinc deficiency and obesity-related miRNAs. Altered miRNAs, including miR-21, miR-22, miR-34a, and miR-130a/b, mediate inflammation, lipid synthesis, and insulin resistance during obesity. ↑ (increase), ↓ (decrease).

The expression of miRNAs in obesity and metabolic diseases, such as miR-21, miR-34a, miR-122, miR-143, and miR-375, have been thoroughly reported. These miRNAs were mainly implicated in metabolic disturbances, such as adipogenesis, insulin resistance, and chronic inflammation (Figure 2). Upregulation of miR-21 and miR-34a during obesity has been shown to have a negative impact on metabolism. These miRNAs target key metabolic regulatory genes *PDCD4* and *SIRT1* [3,37]. The miR-21 downregulation of *PDCD4* leads to increased cell proliferation and decreased apoptosis, resulting in the expansion of adipose tissue and inflammation [28]. The implications for adipose hypertrophy and local and systemic inflammation suggest that miR-21 may represent a potential therapeutic target to reduce adipose expansion and inflammation, although the possible metabolic side effects require further clarification. Continued investigations are needed to identify the factors involved in miR-21 regulation in obese individuals.

Given miR-34a’s involvement in regulating energy metabolism and inflammation, interventions targeting this miRNA may potentially reverse its adverse metabolic effects. Such modulation could inhibit adipogenesis and decrease insulin resistance, offering prospects for long-term metabolic improvement even after weight loss. miR-122, expressed mainly in the liver, targets and regulates lipid synthetic enzymes to manage the production of cholesterol and fatty acids. Elevated levels of miR-122 are reported to correlate with nonalcoholic fatty liver disease (NAFLD) and hyperlipidemia in obese subjects [38,39]. Although these data are promising, it remains unclear what pathways miR-122 modulates in order to control lipid metabolism and if the function of miR-122 differs between different stages of hepatic lipid metabolism.

The finding of downregulated miR-130a and miR-130b expression in the plasma and abdominal adipose tissue of obese people compared to controls is not surprising, as the effect of these miRNAs on pre-adipocyte and macrophage proliferation in vitro suggests an anti-adipogenic role. The reduction in miR-130a and miR-130b expression is consistent with the enhanced lipogenic rate in obese subjects compared to lean subjects [3,40]. The mechanism by which obesity reduces miR-130a and miR-130b, and in what tissues they are expressed, warrants further investigation.

miRNA modulation is tissue-specific, and it is not surprising that obesity-related miRNAs exhibit different regulation among various metabolic tissues. Experimental models revealed that miR-34a is highly upregulated in the adipose tissue of obese subjects compared to lean subjects. The increase in miR-34a expression in adipose tissue inhibited M2 macrophage polarization to the M1 phenotype and subsequently promoted adipose tissue chronic low-grade inflammation, which is one of the major pathological causes of metabolic syndrome [41,42]. Furthermore, in vivo evidence revealed that miR-34a knockout mice developed less glucose intolerance and insulin resistance than their wild-type counterparts, even under the challenge of a high-fat diet, suggesting that miR-34a can exacerbate insulin resistance and glucose intolerance in obesity. It remains unknown if it is possible to achieve localized miR-34a suppression in adipose tissue. It also remains unknown if adipose-targeted interventions are more beneficial than systemic modulation due to possible negative side effects from the global alteration of miR-34a expression levels [28].

The correlation of visceral fat miR-34a with systemic insulin sensitivity in a human population also emphasizes the physiological significance of miR-34a as a novel therapeutic target in obesity and related metabolic diseases. Additional evidence suggested that miR-34a plays a regulatory role in modulating polarization of M2 macrophages and consequently exacerbates insulin resistance in adipose tissue by targeting Klf4, a master transcription factor of M2 macrophage polarization [28,43]. The mechanism underlying this interaction should be further investigated. Based on in vitro results, we speculate that local and partial miR-34a inhibition can induce partial switching from the pro-inflammatory M1 to the anti-inflammatory M2 phenotype, which ultimately attenuates adipose tissue-associated insulin resistance without complete suppression of the inflammatory response in adipose tissue and other peripheral tissues. However, this hypothesis requires more study to determine if adipose-specific partial regulation of miR-34a can be translated into human patients to prevent or alleviate insulin resistance in obesity.

miR-144-3p expression in the plasma has been found to be significantly increased in obese individuals with a high urinary zinc concentration, as a result of environmental zinc exposure. Increased miR-144-3p was significantly associated with both an increase in the homeostatic model assessment of insulin resistance (HOMA-IR), which indicates a decrease in insulin resistance, and a decrease in Hct, hemoglobin, and white blood cell (WBC), respectively, compared to the levels of those without high urinary zinc [44]. Furthermore, the dose–response relationship between miR-144-3p and urinary zinc also suggests that increased zinc may be related to impaired antioxidant defense through increased miR-144-3p and downregulated Nrf2. These findings suggest that it is crucial to maintain a proper amount of zinc intake based on an individual’s metabolic/genetic status in order to balance insulin resistance and oxidative stress [34].

Since increased zinc exposure impaired insulin resistance through increased miR-144-3p and impaired antioxidant defense via decreasing Nrf2. However, zinc is essential for the body’s physiological functioning and plays critical roles in immunity, enzyme activity, metabolism, and homeostasis. Zinc deficiency can impair multiple physiological functions and has been linked to many diseases. Although high-throughput studies have found several miRNAs linked to obesity, little is known about how zinc levels regulate miRNAs, which suggests a potential missing link in understanding metabolic disorders. By modulating Nrf2 signaling, the miRNA miR-144-3p regulates antioxidant gene transcription [34,45].

In individuals with increased BMI and metabolic disorders, higher methylation percentages were observed at the CpG site of the *TAPBP* gene as compared to controls. When comparing individuals with high versus low BMI, multiple loci of the *TAPBP* gene were significantly differentially methylated, especially the gene body, whereas only two loci from *TAOK3* (5’ UTR and TSS200) were differentially methylated at multiple positions. These two genes had similar methylation patterns between high and low BMI, though *TAOK3* displayed higher variability across individuals. These two genes were significantly differentially methylated across subjects with different BMI, indicating that DNA methylation changes in certain regions may be related to BMI variation and that epigenetic mechanisms in some genes may be more related to certain tissues of obese individuals [22].

Evidence also showed that several epigenetic changes, including DNA methylation patterns, can change with an altered level of body weight. These changes play a potential role in regulating different traits of obese people. However, these changes seem to be sex-specific. For example, in offspring from an individual with obesity, most genes with differences in methylation were female-specific [22]. The presence of exosomal miRNAs circulating in human and rat circulation provides evidence for their potential impact on systemic metabolic communication in obesity. Two exosomal miRNAs, miR-148b and miR-23b, enriched in the rat-adipose tissue secretome in comparison with circulation, suggest that the adipose-tissue contributes to systemic metabolic regulation via extracellular communication. Pathway analysis reveals that these exosomal miRNAs target several relevant metabolic signaling networks, including TGF-β and Wnt/β-catenin. The ability to selectively package specific miRNAs into secreted exosomes highlights a novel pathway through which adipose tissue could propagate changes in other cells and tissues to drive the onset of obesity complications [26,46].

In response to the impact of diet-induced weight loss in obese participants, several miRNAs were normalized with altered glucose homeostasis. Specifically, there was a significant and transient decrease in the post-intervention fasting level of glucose from pre-intervention. Circulating miR-122 and miR-21 significantly decrease with the decrease in fasting glucose in obese women only. This association was supported by previous literature showing that these miRNAs may have roles in the development of obesity and T2D. When combined with other known parameters like fasting glucose and miR-122 and miR-21 levels, several dietary-dependent miRNAs could improve a more accurate outcome [27].

These results demonstrate that several miRNAs can be normalized in plasma and adipose tissue within weeks after a change in diet and with weight loss. Thus, many metabolic changes might be influenced via these pathways. In conclusion, the expression of miRNAs are crucial in regulating several metabolic diseases in relation to obesity.

## 4. Zinc-miRNA Interactions

**Figure 3:**
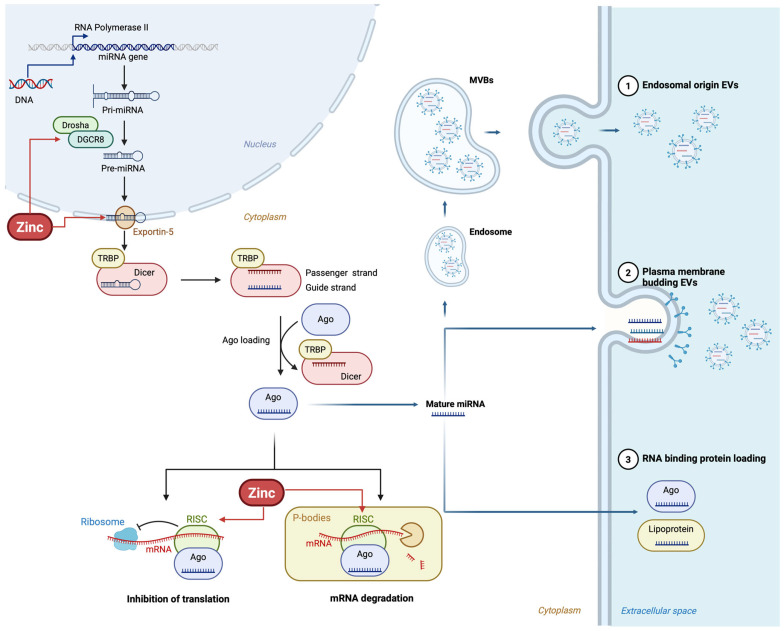
Zinc regulates miRNA biogenesis at multiple steps, including Drosha and Dicer activity, Exportin-5 transport, and RISC complex formation, influencing mRNA translation and degradation.

Zinc plays a key role in the regulation of microRNA (miRNA) expression (Figure 3), with deficiency driving several changes in the expression of miRNAs that impact inflammation and carcinogenesis [2]. Studies have found that zinc deficiency increased pro-inflammatory and oncogenic miRNAs such as miR-21, miR-31, and miR-146a in esophagus and tongue tissues by suppressing tumor suppressor genes *PDCD4* and *PPP2R2A* [5,47]. Although the mechanisms are very convincing and could be considered universal, it is not known whether similar zinc/miRNA interactions contribute to exacerbate systemic chronic inflammation in obesity.

In the animal model of head and neck cancer, zinc repletion reversed the expression of miR-21 and miR-31 after dietary supplementation of zinc, decreasing tumors in zinc repleted mice [5]. Although these results are very interesting, and it has been demonstrated that zinc acts on the pathways of many illnesses, there is no available information whether the changes in the zinc-controlled miRNAs are reversible in human obesity and whether their target molecules involved in processes like adipogenesis and insulin resistance could be affected in a positive way.

The cell-type specificity of the effect of zinc on the miRNAs adds further complexity to this regulation. miR-31 levels are mainly in tumor cells in the zinc-deficient state. Conversely, miR-21 is mainly in stromal cells, not in tumor cells [5,48]. These zinc-induced changes in miRNAs may have multiple consequences dependent on cell type, suggesting that zinc’s action over miRNAs could produce diverse or opposite metabolic effects in vivo depending on specific cell targets within the same tissue/organ. Considering that the effect of zinc on miRNA regulation is cell-type-specific, would zinc modulate miRNA expression in adipocytes, macrophages and other fat tissue-resident cells similarly or differentially.

Although in the field of miRNA studies in obesity, the possibility of dietary modulation of epigenetic regulators of gene expression has been studied; for the intervention by zinc supplementation to be effective and successful in individuals, it is essential that zinc is absorbed and transported effectively to reach the target tissues. For example, individual differences in zinc absorption, metabolism, and inflammatory status may affect the efficacy of zinc intervention through miRNAs. One possibility is to create nutrition systems incorporating both individual’s predispositions and genes responsiveness to zinc dietary treatments based on miRNAs. However, further studies of individuals are necessary, as reliable markers of predicting zinc responsiveness need to be validated.

Circulating miRNAs are highly stable in the circulation of humans; levels of some miRNAs decreased during dietary zinc restriction and returned to pre-restricted values with zinc repletion [2]. Levels of the circulating miRNAs, miR-10b, miR-145, and miR-155, significantly decreased during zinc depletion, indicating that the responses of these miRNAs can occur acutely with dietary depletion and may be used as indicators of dietary inadequacy [49]. All plasma miRNA measurements restored with zinc repletion. Even though the results are encouraging, in several other published plasma/serum profiling studies in zinc-deficient groups (e.g., pregnant women, patients with acute lung injury), no correlations between zinc status and the miRNAs have been described, and further studies on the role of zinc on circulating miRNAs and their correlation with zinc plasma levels and zinc status would be useful.

Because the changes in miRNA occurred acutely, determining miRNA kinetics over time, and profiling miRNAs at different stages of zinc depletion may be relevant. Some miRNAs were modulated in all phases, and other miRNAs appear to be changed in certain stages but not others of zinc depletion [2]. Studies are required to explore whether this type of miRNA modulation would also occur during zinc depletion in humans and whether the changes in circulating miRNAs were to be translated as immediate improvements to the metabolic syndrome, or that chronic dietary zinc deficiency would cause irreversible damage to the miRNA regulatory machinery [50].

Zinc excess has been linked to altered miRNA expression. Paradoxically, zinc supplementation enhanced miR-144-3p expression [34], leading to downregulation of the antioxidant transcription factor Nrf2. This is in contrast with several lines of evidence that indicate a favorable role of zinc in metabolic syndrome or obesity. This could have great biological implications because zinc would be affecting negatively to Nrf2 pathway, which also controls antioxidant levels in different illnesses.

The interaction between miR-144-3p and Nrf2 pathway might reflect the involvement of zinc in the mechanism by which dysregulation of zinc homeostasis and/or metabolism may contribute to the progression of metabolic diseases such as diabetes and obesity [34]. Although several human studies indicate that impaired zinc status might contribute to the pathophysiology of type 2 diabetes, it also remains possible that other as-yet-undefined zinc responsive signaling pathway(s) could modulate metabolism in either a beneficial or harmful way, according to zinc dose and administration mode.

Several dietary factors modulate the regulation of miRNA. One of these is resveratrol, a dietary polyphenol. As an example, resveratrol downregulated the levels of pro-inflammatory miRNAs like miR-21 and miR-146a [51], and also modulated the levels of Nrf2, the target of miR-144-3p. These are the same miRNAs that are targets for zinc. Since zinc also regulates miRNA levels, it is possible that it interacts with the same regulatory pathways to coordinate metabolic inflammation. These results highlight the possibilities for further exploring synergistic dietary strategies that may affect different regulatory mechanisms of the same miRNAs to treat metabolic disorders.

The interactions of different nutrients and minerals on the regulation of miRNAs are largely unexplored. Given the diverse health functions attributed to dietary bioactive compounds such as zinc, resveratrol, and fatty acids, there is a great need to investigate their impact in obesity prevention and treatment. The evidence indicates that various nutrients and dietary bioactive compounds influence miRNA expression and metabolic function in an intricate manner; this needs to be more thoroughly studied and understood in humans to identify specific dietary and lifestyle strategies to improve outcomes in obesity.

**Figure 4:**
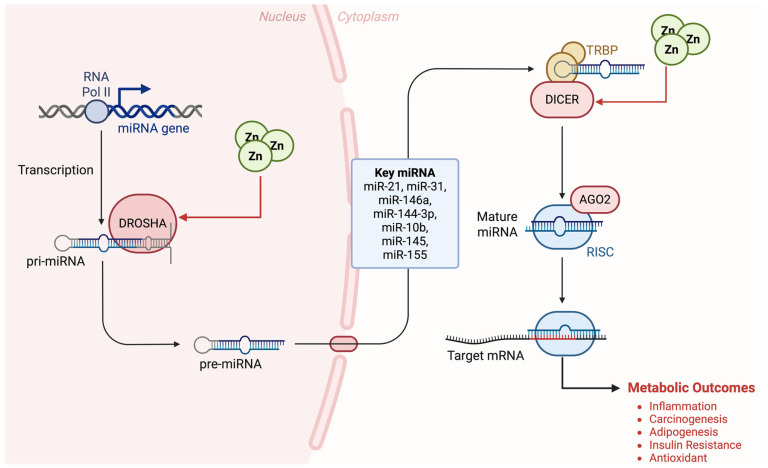
The zinc–miRNA axis in metabolic regulation. Zinc modulates Drosha and Dicer function, affecting key miRNAs and their target mRNAs, leading to metabolic outcomes such as inflammation and insulin resistance.

Taken together, the results of several researchers strongly support a linkage between zinc-responsive miRNAs such as miR-21, miR-31, miR-146a, and miR-144-3p, and the etiology of metabolic diseases such as obesity [2,5,34,47,51], suggesting that specific miRNAs play important regulatory roles in adipogenesis, inflammation, insulin resistance, and perhaps more (Figure 4). Studies on other regulatory signals, the bioavailability of dietary zinc, and the gene expression of zinc transporters in patients are needed.

Previous work on the mechanistic role of zinc and miRNA in the metabolic function has revealed that zinc is associated with biogenesis, processing, and function in general of miRNAs; most of them require zinc, and zinc deficiency or excess may modify their function and activity. The zinc-sensitive biogenesis and metabolism may play an important role as a pathophysiologic signal and can be used as a molecular target [4]. Thus, we suggest that changes of zinc-dependent miRNAs could contribute to early and/or intermediate stages of metabolic dysfunction. These findings might have clinical implications as the identification of zinc-dependent miRNAs that would alter adipogenesis, insulin resistance, or any other metabolic function would open new opportunities for diagnosis and therapy.

## 5. Clinical Applications

**Figure 5:**
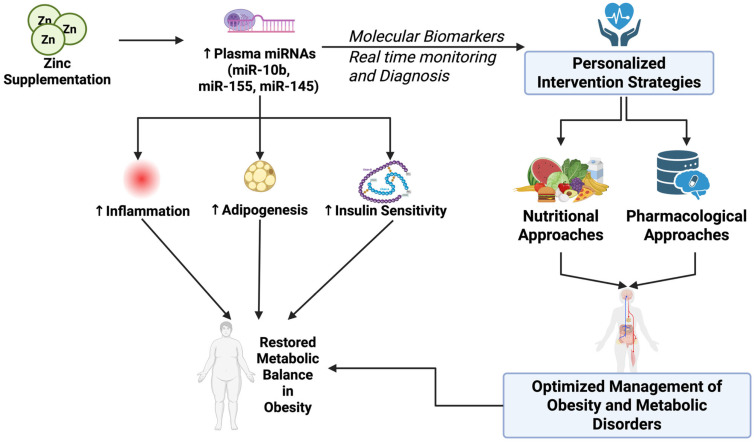
Zinc supplementation restores metabolic balance by modulating plasma miRNAs and enabling personalized nutritional and pharmacological intervention strategies in obesity. ↑ (increase), ↓ (decrease).

Advances in diagnostic and therapeutic strategies are increasingly focusing on the integration of molecular biomarkers and personalized interventions to combat obesity and metabolic disorders (Figure 5). Exploiting zinc-sensitive miRNAs as promising novel diagnostic and therapeutic approaches allows for real-time and accurate monitoring and treatment of metabolic syndrome by optimizing nutritional and pharmacological intervention. Therefore, as part of our contribution in comprehending the novel mechanisms of zinc for treating obesity and metabolic disorders, we attempt in this section to discuss current and developing clinical applications via biomarkers for prevention of metabolic syndrome based on personalized diagnosis.

### 5.1. Biomarker Development

New data have emerged demonstrating the potential utility of circulating miRNAs as dynamic biomarkers to assess the real-time changes of zinc status following dietary interventions. Dietary studies in healthy adult males have identified a series of plasma miRNAs (miR-10b, miR-155, miR-200b, miR-296-5p, miR-373, miR-92a, miR-145, miR-204, and miR-211), which are repressed during zinc depletion and activated following zinc repletion [2,49]. The reversibility of these miRNAs in dietary zinc intervention confirms their potential as biomarkers for longitudinal follow-up in clinical trials of lifestyle/clinical interventions to control obesity. The impact of miRNAs on inflammatory, oxidative, and metabolic processes reinforces their connection to obesity pathogenesis. Although the ability of miRNAs to be reversibly regulated by zinc suggests promise in this area, additional validation is required to examine the association between zinc repletion, miRNA changes, and health outcomes.

The advantage of circulating miRNAs as biomarkers is their ability to indicate the status of early/subclinical zinc depletion, which cannot be easily detected with biochemical markers of zinc nutritional status. The reversibility of the selected miRNA signature associated with zinc depletion/repletion suggests an added value of using circulating miRNAs as longitudinal biomarkers to monitor the efficacy of clinical interventions in metabolic disorders, such as obesity and insulin resistance [52]. Furthermore, given the functional effects of miRNAs and their contribution to zinc-associated metabolic disorders, these miRNAs can be applied for risk stratification to predict an individual’s metabolic response toward dietary zinc supplementation. To achieve this objective, there must be continued studies to validate the utility of miRNAs as biomarkers across varying metabolic conditions and to establish universal standards of miRNA profiling to facilitate comparisons.

For example, circulating and exosomal miRNAs, like miR-21 and miR-122, have been found to be responsive to weight-loss interventions in obese individuals [27], supporting their potential as early, real-time biomarkers for the identification of individuals with a greater potential to achieve metabolic improvement in lifestyle interventions to ameliorate metabolic disturbances associated with obesity and insulin resistance. To better implement the use of circulating and exosomal miRNAs as real-time and robust biomarkers, validation studies that integrate large clinical trials that directly correlate specific miRNA changes with physiological changes (reduced inflammation and improvements in lipid metabolism, among other relevant indices) are necessary.

It is known that some circulating miRNAs also respond quickly to changes in dietary interventions and exert mechanistic effects involved in improved metabolism [2]. As such, these miRNAs may be suitable targets to determine real-time responses associated with zinc intervention. The possibility of targeting specific miRNAs for therapy is not as likely with the other traditional biochemical measurements used to examine the nutritional status of zinc, due to their lack of mechanistic roles in driving improved metabolism during zinc intervention. However, future research is needed to determine whether these circulating and exosomal miRNAs have a causal effect on metabolic changes observed during dietary intervention to establish their utility as diagnostic biomarkers or possible therapeutic targets. Further studies are also needed to fill the gap of crosstalk among tissues during dietary interventions. 

Observational studies have documented a correlation between plasma zinc and altered expression of circulating miRNAs, such as miR-191-5p, miR-188-5p, miR-145-5p, and miR-143-3p [53]. Interestingly, these miRNAs are also involved in the insulin signaling pathway and glucose metabolism, supporting their potential as a functional proxy of zinc status in association with metabolic disorders. These correlations could be used to develop an miRNA diagnostic tool to stratify individuals depending on their phenotypic zinc and metabolic responses. It must be pointed out that there is substantial inter-individual variation, highlighting the need for large-scale studies to validate the use of circulating miRNAs for nutritional diagnostic, prognostic, or therapeutic purposes.

Furthermore, miR-144-3p has also been documented to regulate Nrf2, resulting in increased oxidative stress and exacerbated insulin resistance [34]. These studies demonstrate that, in addition to circulating miRNA as potential biomarkers, it is also important to consider functional miRNAs with causal regulatory relationships. The utilization of mechanistic findings would allow for more targeted therapies, such as altering zinc supplementation to specifically modulate the expression and activity of key miRNAs involved in metabolic regulation. In contrast, due to the differential effects of zinc homeostasis on metabolism as well as the potential adverse effects of both deficiency and excess, such targets could not be utilized. 

During dietary intervention, the dynamic changes in circulating miRNAs were associated with changes in inflammatory makers (e.g., CRP and IL-6) [54]. Therefore, the combination of circulating miRNAs as biomarkers with the conventional assessment (biochemical measurements), as well as inflammatory markers, can enhance the diagnostic accuracy and reliability. It must be acknowledged, however, that the development of a standardized circulating miRNA biomarker panel in nutritional research and for clinical application requires careful considerations. Biomarker panels must be designed to target various pathological processes and accurately capture the dynamic changes in diseases, and this process would be difficult to achieve when dealing with complex metabolic pathways.

Studies in mouse models of obese and zinc-deficient/supplemented conditions identified that two miRNAs, miR-27a and miR-103, are expressed in response to nutritional or metabolic status [55], and these miRNAs also mediate adipogenesis, inflammation, and zinc deficiency. These findings suggest an improved approach that utilizes circulating miRNAs, which may lead to enhanced effectiveness and predictive value to monitor the progress of obesity progression and/or interventions. In the long term, these changes would be needed to validate if the newly proposed zinc-status-responsive circulating miRNAs have clinical utility. Given the nature of intervention studies that are usually carried out at moderate and tightly controlled conditions, a high prevalence of clinical effectiveness or significance may be seen, which may not be realistically translated to the population in broader, real-life conditions. However, miRNAs such as miR-27a and miR-103, responsive to both nutritional and metabolic status, may be the key mediators in monitoring the effectiveness of nutrition-based clinical trials for obese populations, especially when there may be a need to tailor the intervention toward a particular subpopulation [56].

In conclusion, circulating miRNAs represent a type of promising biomarker. In the future, the integration of a multi-omic approach to investigate complex networks of miRNAs and nutrients in nutritionally and metabolically relevant tissue samples would advance the field and develop practical means for clinical and public health applications. The identification and functional validation of the direct, specific interactions between miRNA genes and nutrient metabolism would lead to a new era of biomarkers and therapeutics that focus on individualized diagnosis and prevention/treatment strategies. This strategy fits with ‘nutrimiromics’ an emerging area of research on nutrition, miRNAs, and health. Nutrimiromics is an interdisciplinary approach to nutrient regulation of miRNA expression in dynamically changing environments and in specific tissues involved in the etiology of metabolic diseases. In response to nutrients, miRNAs can regulate metabolic, inflammatory, and epigenetic pathways to influence human metabolism and, therefore, overall health outcomes. In addition, clinical trials can utilize personalized treatment plans in order to tailor an appropriate dietary and lifestyle approach to minimize the impact of risk factors (e.g., age, obesity, etc.) on metabolic diseases.

### 5.2. Treatment Strategies

Zinc supplementation can also restore some of the miRNA that were dysregulated during zinc deficiency. In one human intervention study, zinc repletion quickly increased plasma levels of miR-10b, miR-155, and miR-145, which are suppressed during dietary zinc deficiency [49]. While these miRNA are involved in the regulation of inflammation, adipogenesis, and insulin signaling pathways, the current evidence does not reveal how zinc dose, duration, and individual genetic factors interact to affect miRNA restoration. This suggests that future human research should explore the degree to which these factors contribute to the effectiveness of zinc supplementation for restoring zinc-sensitive miRNAs to optimal levels. 

Another point to note is the specificity in the tissue response to zinc-responsive miRNAs. Normalizing miR-155 following zinc repletion has, for example, been shown to regulate inflammatory pathways in metabolic tissues [2,57]. This response could then translate into changes in tissue function, but whether this phenomenon is sustained or not requires future investigation. More research is needed on this response, as the recovery of certain zinc-responsive miRNAs, like miR-155, might induce homeostatic changes for other miRNAs involved in inflammation. The effectiveness of zinc intervention depends on how it restores miRNA regulation in particular tissues.

Under conditions of chronic zinc deficiency, zinc supplementation has also been shown to reduce pro-inflammatory and oncogenic miRNAs, such as miR-21 and miR-31. These miRNA target several tumor suppressor genes, including *PDCD4* and PPP2R2A. Interestingly, zinc can reduce the expression of both miR-21 and miR-31 [5,58]. The reversibility of miRNA signatures by zinc offers the chance to use this trace mineral to limit inflammation-driven cancer and other diseases with metabolic links, but the relevance of these findings to the chronic low-grade inflammation present in obese individuals warrants further research. Such studies could investigate the utility of miR-21 and miR-31 downregulation as a means of treating chronic inflammation in obese people. Also, the possibility of off-target effects of zinc on other miRNAs should be investigated to determine the overall safety of this supplementation [59].

The influence of zinc on miRNA expression and cancer development highlights the general usefulness of nutrition in mediating inflammation via changes to the levels of certain molecules such as miRNA. Both human and animal studies showed that zinc could improve immune homeostasis and decrease the molecular signs of chronic inflammation by regulating gene expression, including miRNA [5,60]. Still, what the exact influence of zinc is on the complex network of nutrients, hormones, and inflammatory molecules in the obese must be elucidated, to determine if it can be utilized for precision nutrition interventions.

In clinical trials, the levels of zinc metalloproteins, such as MT1G and ZIP-14, decreased with zinc supplementation, but plasma inflammatory cytokines did not. The findings indicate that the anti-inflammatory action of zinc supplementation is intracellular in nature, including miRNAs [61]. When intracellular zinc is restored through supplementation, transcriptional and post-transcriptional regulatory pathways are activated in multiple tissues. In this way, intracellular zinc and associated signaling pathways can prevent or treat inflammation in disease. The results suggest that the supplementation effect on clinical outcomes is mediated by a mechanism other than cytokine changes. Clinical intervention trials focusing on the changes in zinc homeostasis with zinc supplementation combined with miRNAs could lead to a better understanding of the complex mechanisms of zinc supplementation. Investigating intervention studies across populations with varying basal inflammatory conditions will further elucidate the action of zinc in metabolic inflammation. However, excess zinc exposure also altered miRNA expression patterns. Excessive zinc exposure increases the abundance of miR-144-3p in the cells and induces apoptosis. This miRNA targets Nrf2, thereby impairing oxidative defenses and increasing insulin resistance [34,50].

In humans, too much zinc will cause a multitude of biological functions that, in turn, may harm the body. Thus, it is very important that intake is neither too high nor too low. The reversibility of miRNA with zinc highlights the opportunity to supplement this trace mineral in order to maintain an optimal state in inflammatory regulation and metabolism and ultimately the individual’s overall health. It also suggests that miRNAs are dynamic biomarkers responsive to dietary intervention and zinc metabolism [56,62]. Future research should seek to find zinc intake thresholds at which zinc can provide beneficial effects to obese and chronically inflamed individuals by optimizing the expression of miRNAs.

However, as mentioned before, high zinc intake induces miRNAs that downregulate key proteins in energy metabolism. Zinc supplementation decreased miR-144-3p in cells and increased Nrf2. Excessive zinc supplementation negatively regulates Nrf2 through miR-144-3p, leading to increased oxidative stress and glucose intolerance [34]. It is plausible to think of this relationship as a U-shaped dose–response curve where zinc must be kept within optimal thresholds to ensure its efficacy and safety.

Also, the analysis of the miRNA levels during clinical contexts could aid in preventing adverse responses induced by zinc exposure, allowing a more effective use of zinc as an intervention for different conditions. The incorporation of zinc for therapeutic purposes would benefit from individualized supplementation strategies that account for underlying genetic and inflammatory profiles, including miRNA [51,56]. However, this type of approach will require further work from an analytical perspective (e.g., improvement and standardization of techniques that measure miRNA in clinical and laboratory samples), as well as overcoming difficulties and challenges in implementing personalized approaches in population health nutrition. In this context, it is imperative to acknowledge other key players, such as dietary components, in orchestrating inflammatory pathways involving miRNAs. For example, the polyphenol compound resveratrol can lower miR-21 and miR-146a abundance [51]. These two miRNAs participate in inflammation, oxidative stress, and insulin sensitivity. Thus, while zinc influences the abundance of miRNAs that play a central role in the metabolism and immune system, its effects should be discussed within the context of more complex nutritional and physiological systems. Several dietary nutrients, besides zinc, can also alter the abundance of miRNA and thereby exert beneficial effects in health. More studies need to address the interaction and the importance of these molecules in the development and progression of metabolic inflammation, to determine their application in anti-obesity therapies.

Recent research indicates that dietary zinc regulates miRNAs that mediate insulin signaling pathways and contribute to glucose homeostasis. Genomic and proteomic data showed that zinc homeostasis in pancreatic cells is maintained by several miRNAs [63,64]. Future human trials should determine whether incorporating other nutrients that regulate common miRNAs may synergistically reduce inflammation and provide enhanced metabolic benefits. It is well known that diets induce expression of miRNAs related to insulin resistance, and this recent study is yet another proof of this. These effects suggest the use of zinc and other dietary bioactives to target miRNAs that modulate glucose metabolism and insulin resistance to treat diseases associated with insulin resistance. However, the findings of this study are limited by the time-point measure, because future studies should consider the longitudinal aspects of zinc supplementation in combination with other diet therapies.

Recent molecular evidence underscores the necessity to target the chronic low-grade inflammation in individuals with obesity to effectively combat the disease. There have been many new discoveries in the molecular obesity field lately in signaling pathways and key players [65]. Zinc’s role in miRNA regulation could be investigated as part of larger approaches. Such an approach, as a starting point, must explore the individual variation present in obesity at the levels of miRNAs that are involved in zinc supplementation [66]. The role of these miRNAs and genes in obesity needs to be further investigated in both animal and human models.

The newly found role of miRNAs as either biomarkers or players in zinc’s impact on health emphasizes the need to assess this complex interaction from the perspective of a nutrigenomics approach. These miRNAs are pivotal for mediating the effects of zinc on inflammation, cellular zinc metabolism, cell survival, and proliferation, and they also influence cancer biology by either inhibiting or stimulating tumors. miR-21, -31, -146a, and -144-3p are zinc-sensitive miRNAs involved in multiple pathways and have been shown to have similar patterns of expression during zinc exposure in certain contexts [2,34,47,51].

The connection between zinc and miRNAs still lacks more investigation in a broader context of the obesity spectrum. Future studies must aim to understand the mechanisms and regulation of zinc and miRNA in obesity pathogenesis, with a more individualized intervention strategy. 

In sum, several lines of evidence suggest zinc plays an essential role in the modulation of specific miRNAs in obesity. Although a large number of studies have shown that zinc can be an effective treatment for obese individuals, the evidence from the studies above underscores a paradoxical role of the trace mineral. The findings suggest that both zinc deficiency and zinc excess can alter the expression of these miRNAs, leading to the development of chronic inflammation. The next step should be to explore human populations and to determine the effective zinc range for human health in the obesity spectrum by utilizing the available tools of miRNAs.

## 6. Future Perspectives

The integration of multi-omics approaches will greatly contribute to the understanding of the crosstalk between zinc, miRNAs, and metabolic profiles of obesity [67]. In addition, application of these technologies may enable the identification of novel zinc-sensitive miRNAs and target networks that can further define zinc’s regulation in metabolic health. Multi-omics includes several disciplines, such as genomics, epigenomics, transcriptomics, proteomics, and metabolomics. Integrating transcriptomic or proteomic data together with miRNA profiling may define zinc’s action on adipogenesis and insulin sensitivity [8,68]. However, due to the complexity of interpreting high-dimensional datasets, novel models of multifactorial interactions among various factors will be required. To overcome the variability observed in the effects of zinc supplementation across different populations, systems biology and computational modeling approaches will be necessary. Application of multi-omics approaches would be invaluable to identify novel zinc-sensitive miRNAs as well as their effects on protein levels and metabolite profiles, which are associated with metabolic diseases [8]. Application of longitudinal studies is further required to establish dynamic correlations between zinc-responsive miRNAs and clinical outcomes, such as in response to dietary intervention. As an example, transcriptomics and metabolomics analysis could be paired to investigate how zinc regulates lipid metabolism through miRNAs [52,69]. Since there is currently limited data from longitudinal studies that examine links between zinc status, miRNA expression, and clinical outcomes, additional investigation may clarify zinc’s mechanistic actions on metabolic processes via miRNAs and provide potential approaches for intervention.

For prediction and mechanistic modeling, systems biology tools are necessary to interpret the integration of zinc-sensitive miRNAs into biological pathways, which will define zinc regulation of metabolic diseases. These computational models will allow researchers to describe individual differences to predict the response of patients to zinc interventions in obesity [8]. These tools should also allow interpretation of high-throughput data from multiple layers of regulation to achieve predictive and personalized modeling. Additionally, significant challenges and new techniques for bioinformatics analyses will need to be considered for systems-based application. Therefore, computational modeling and a systems-based approach will expand the translational impact of experimental findings into the clinic.

More research is warranted to establish longitudinal correlations in clinical cohorts between zinc-sensitive miRNAs and metabolic health outcomes. These observations will allow more effective nutritional interventions. Capturing the long-term dynamics between zinc and disease progression in patients will allow the application of effective nutritional intervention with zinc. These studies may need to be conducted in a cohort with appropriate design, data, and standards for the implementation of clinical application.

The gut microbiome in regulating zinc absorption as well as its action on miRNAs is a major contributing factor for variable response to zinc treatment in different phenotypes of obesity [8,70]. The composition and functionality of the microbiome modulate zinc bioavailability, indirectly affecting systemic miRNA expression. However, the relationship between the microbiome and miRNAs that regulate zinc uptake is not clear. Gut metagenomics should be paired with miRNA profiling to reveal the mechanisms by which microbiome-mediated zinc bioavailability affects miRNAs and, therefore, metabolic health. The zinc-miRNA networks in relevant tissues of obesity, such as adipose, liver, and skeletal muscle, are not well described [45,71]. The characterization of zinc-miRNA networks in specific tissues of obesity is crucial because it will reveal tissue-specific effects for zinc in regulating gene expression, leading to better interventions that address obesity and related metabolic abnormalities. For example, tissue-specific responses for miR-21 expression have been found in zinc-deficient adipocytes versus hepatocytes. Therefore, more research is warranted to assess zinc-miRNA networks across tissues for precise targeting of intervention to specific tissues.

Advanced in situ profiling may enable the discovery of interactions and changes within heterogeneous tissue environments, and single-cell sequencing technology has recently emerged as a tool to dissect the regulation of gene expression in complex tissues. Such technologies allow spatial and temporal relationships to be identified in miRNA–zinc interactions that are dependent on the specific cell type and in different tissues. Applying these technologies is capital-intensive, but it can enhance our understanding of the link between cellular context and specific regulatory pathways that may impact metabolic adaptations [72,73]. The variability of zinc concentration and expression of zinc transporters in tissues may elicit different miRNA responses to zinc deficiency or treatment across tissues and, therefore, contribute to the controversy regarding zinc’s effects on metabolic diseases. Understanding how differential zinc distribution and metabolism among tissues may affect miRNA activity and their roles in metabolic processes in obesity is required to address the variability in response to zinc treatment among different populations [1,74,75].

In regard to future applications for diagnosis and clinical treatment with miRNAs to address zinc status in obesity, emerging evidence that zinc modulates circulating levels of miRNAs, such as miR-10b, miR-145, and miR-144-3p, indicates the potential for these miRNAs to act as valuable and clinically applicable biomarkers to diagnose and assess the effectiveness of zinc intervention [2,34]. These circulating miRNAs showed changes in response to zinc interventions in obesity. However, to make this research applicable to the clinic, the use of a panel of circulating miRNAs must be standardized among clinics. Clinical testing of biomarkers is often limited because of confounding factors, such as sex and age differences in response to treatment, variation in diet, variability in disease phenotypes, or other diseases. Moreover, standardizations for testing require establishment of reference ranges for levels to have clinical impact. Therefore, large studies and patient samples with well-documented cohorts are necessary to standardize clinical use for biomarkers.

Large and comprehensive cohort studies across multiple populations may support a more definitive conclusion about the use of miRNA biomarker signatures in obesity. Such studies will require substantial human and financial resources as well as standardization of protocols and controls. However, this research can yield an effective biomarker panel that could predict zinc supplementation outcomes as well as be widely applicable to a variety of genetic backgrounds and diverse populations [76,77].

Developing models of the combined biomarkers to predict therapeutic benefits is critical for translating research into the clinic. These prediction models can be further enhanced by integrating miRNA biomarker signatures with standard clinical measurements of metabolic health and with other risk factors, leading to improvements of targeted therapies. Overall, this is a critical research topic that will be required to determine the validity of this model in predicting zinc treatment [78,79].

The potential for an miRNA biomarker panel to be able to predict therapeutic benefits underscores its application for diagnosis and potential stratification of individuals into clinical trials. In order to design targeted therapies with proper dosage and frequency of zinc interventions to achieve efficacy, dose–response studies are required. The dose–response is complicated due to inter-individual variation in zinc homeostasis and transporter genotypes that can impact transporter activity [80]. As such, dosing studies that determine the ideal window of therapeutic zinc supplementation that is both beneficial to obesity-related health outcomes but also does not trigger adverse effects are warranted.

Additional research is warranted in determining how the dose, duration, and route of administration influence cellular concentrations of zinc, ultimately altering zinc transporter and protein expression, miRNA biogenesis and processing, and biological function. For example, zinc treatment can induce expression of miR-144-3p, which may play a significant role in impaired oxidative defense and insulin resistance [34,63]. Such a paradox is a potential hazard for zinc treatments because while many beneficial effects have been described, an imbalance of gene regulation can have potential hazards. Future research should focus on precision medicine and nutritional recommendations that are specific to individuals with respect to zinc requirements, genetics, epigenetics, and phenotypes in order to deliver effective intervention that delivers maximal therapeutic benefits without risks. Incorporating zinc and miRNA assessments into study protocols has the potential to allow differentiation of patients who are likely to respond positively or negatively to clinical interventions with zinc supplementation and can allow further personalized nutritional therapies for obesity [70,81]. The success of incorporating the role of the zinc-miRNA pathway and its interactions into clinical protocols will be dependent upon our success in translating results obtained in vitro and in vivo to real-world medical use. This poses significant challenges to the field but presents a viable model by which patients who require personalized and individualized treatment could be selected and treated with the best possible care for obesity.

Although this review integrates mechanistic, clinical, and translational findings, several limitations should be acknowledged. Most available studies are cross-sectional or short-term, limiting the ability to infer causal relationships between zinc status, miRNA expression, and metabolic outcomes. In addition, there is a scarcity of longitudinal and dose–response human studies that evaluate optimal zinc levels or supplementation thresholds required to modulate miRNA profiles effectively. The heterogeneity in study design, population characteristics, and analytical methods further complicates data comparison and meta-analysis. Addressing these limitations through well-designed, long-term clinical trials will be essential to clarify the therapeutic potential of the zinc–miRNA axis in metabolic disease management.

Future studies are needed to identify the interaction of nutrients that play a critical role in miRNA–zinc-mediated benefits in metabolism [8,63]. Many recent studies indicate that a variety of dietary compounds can alter the expression and processing of several miRNAs related to metabolic diseases. As an example, resveratrol downregulates the expression of several pro-inflammatory miRNAs, such as miR-21 and miR-146a [82]. As mentioned earlier, the expression of these two miRNAs is altered by zinc. Therefore, in combined intervention trials, one treatment can enhance or interfere with the effects of another. Therefore, zinc with a specific diet will have variable effects. Zinc may be combined with different nutrients, or the dosage may be lowered or increased as individuals exhibit phenotypic variances that are linked to underlying genotypic backgrounds.

In order to tailor zinc intervention strategies to the unique patient, nutrient supplementation approaches will require an understanding of these interaction networks to effectively address the multiple mechanisms underlying obesity [70,83]. Also, we should understand if one bioactive can interfere with the other, such as one nutrient inhibiting zinc absorption or affecting zinc-mediated miRNA regulation. This research should address the challenges to zinc nutrition and determine the interactions between nutrients on the metabolic effects via miRNA in vivo. In addition to experimental and clinical studies, computational modeling and machine learning approaches hold promise for predicting zinc–miRNA interactions in the context of personalized nutrition. These tools can integrate multi-omics datasets, identify regulatory networks, and potentially forecast individual responses to zinc supplementation. Incorporating such predictive models may facilitate the development of tailored nutritional interventions and improve our understanding of the regulatory roles of zinc in miRNA-mediated metabolic pathways. By filling these research gaps, the field of nutrigenomics can use the knowledge on nutrient-miRNA interactions to design comprehensive and personalized nutritional programs. This research area will pave the way for designing comprehensive precision nutrition plans to tackle obesity.

## 7. Conclusions

Zinc homeostasis is essential for maintaining metabolic health through its regulation of microRNA (miRNA) expression involved in adipogenesis, insulin signaling, inflammation, and oxidative stress. Both zinc deficiency and excess disrupt this balance, altering the expression of key miRNAs such as miR-21, miR-34a, miR-122, and miR-144-3p—molecules that critically modulate metabolic and inflammatory pathways. Zinc repletion has been shown to restore dysregulated miRNAs and improve insulin sensitivity and lipid metabolism, underscoring its reversibility and therapeutic potential.

However, excessive zinc intake may upregulate miR-144-3p, suppress the antioxidant transcription factor Nrf2, and exacerbate oxidative stress, suggesting a U-shaped relationship between zinc status and metabolic health. Therefore, maintaining optimal zinc levels is vital for achieving metabolic balance.

The integration of zinc-responsive miRNAs as biomarkers in clinical and nutritional research offers a promising avenue for precision nutrition strategies. Future studies should focus on elucidating tissue-specific zinc–miRNA interactions, determining safe and effective supplementation thresholds, and validating miRNA signatures for clinical monitoring. Ultimately, understanding the zinc–miRNA axis provides a foundation for developing personalized therapeutic interventions to prevent and treat obesity and related metabolic disorders. The incorporation of nutrimiromics into clinical practice guidelines and public health nutrition frameworks may enable tailored dietary recommendations, early risk detection, and more effective interventions for metabolic diseases in diverse populations.

## Data Availability

No new data were created or analyzed in this study. Data sharing is not applicable to this article.

## Abbreviations

ATP	Adenosine Triphosphate
BMI	Body Mass Index
CpG	Cytosine–Phosphate–Guanine
CRP	C-Reactive Protein
*DGCR8*	DiGeorge Syndrome Critical Region 8
ER	Endoplasmic Reticulum
HDL-C	High-Density Lipoprotein Cholesterol
HOMA-IR	Homeostatic Model Assessment of Insulin Resistance
IL-6	Interleukin-6
LDL-C	Low-Density Lipoprotein Cholesterol
miRNA	MicroRNA
MT1G	Metallothionein 1G
NAFLD	Nonalcoholic Fatty Liver Disease
NF-κB	Nuclear Factor Kappa B
Nrf2	Nuclear Factor Erythroid 2–Related Factor 2
PCR	Polymerase Chain Reaction
*PDCD4*	Programmed Cell Death 4
PI3K/Akt	Phosphatidylinositol 3-Kinase/Protein Kinase B Pathway
PPP2R2A	Protein Phosphatase 2 Regulatory Subunit B Alpha
qRT-PCR	Quantitative Reverse Transcription Polymerase Chain Reaction
RISC	RNA-Induced Silencing Complex
ROS	Reactive Oxygen Species
*SIRT1*	sirtuin 1
T2D	Type 2 Diabetes Mellitus
*TAOK3*	TAO Kinase 3
*TAPBP*	TAP Binding Protein
TG	Triglycerides
TGF-β	Transforming Growth Factor Beta
VEGF	Vascular Endothelial Growth Factor
WBC	White Blood Cells
ZIP	Zrt- and Irt-like Protein
ZnT	Zinc Transporter
